# Oxidative Stress Markers in Urine and Serum of Patients with Bladder Cancer

**DOI:** 10.3390/antiox12020277

**Published:** 2023-01-26

**Authors:** Sabina Galiniak, Mateusz Mołoń, Marek Biesiadecki, Agnieszka Mokrzyńska, Krzysztof Balawender

**Affiliations:** 1 Institute of Medical Sciences, Medical College, Rzeszow University, 35-310 Rzeszow, Poland; 2 Department of Biology, Institute of Biology and Biotechnology, Rzeszow University, 35-959 Rzeszow, Poland

**Keywords:** oxidative stress, urogenital cancers, urothelial carcinoma

## Abstract

Oxidative stress is defined as an imbalanced state of the production of reactive oxygen species and antioxidant capacity that causes oxidative damage to biomolecules, leading to cell injury and finally death. Oxidative stress mediates the development and progression of several cancer diseases, including bladder cancer. The aim of our study was to determine markers of levels of the oxidative stress in serum and urine in the same patients in parallel in serum and urine. Furthermore, we tried to estimate the associations between oxidative stress markers and the type of cancer, its clinical stage and grade, as the well as correlations between serum and urinary markers in patients with bladder cancer. Sixty-one bladder cancer and 50 healthy volunteers as a control group were included. We determined the serum and urine levels of advanced oxidation protein products (AOPP), Amadori products, total antioxidant capacity, total oxidant status (TOS), oxidative status index (OSI), and malondialdehyde. We confirm that almost all markers are elevated in serum and urine from patients with bladder cancer than from healthy subjects. Moreover, we did not find differences in the level of oxidative stress markers and the type of tumor, its clinical stage, and grade. We noted correlations between serum and urinary biomarkers, in particular TOS and OSI. Our results clearly indicate the participation of oxidative stress in the development of bladder cancer.

## 1. Introduction

Bladder cancer is the seventh most frequently diagnosed cancer in the world for men, while when both sexes are taken into account, it falls to a tenth. The world age standard (per 100,000 people/year) incidence rate is 9.5 for men and 2.4 for women [1]. Regarding mortality, bladder cancer is the 13th cancer most deadly worldwide, with the bladder cancer age-standardized mortality rate (per 100,000 people/year) 3.3 for men versus 0.86 for women [1]. Unfortunately, the number of new cases continues to increase, especially in developed countries [2]. Bladder cancer has a complex and multifactorial pathophysiology. Numerous risk factors are involved in the development of urothelial cell cancers [3,4]. The most important factors for bladder cancer include active and passive smoking, accounting for about 50% of cases [5]. In addition, the second most important risk factor for bladder cancer is occupational exposure to aromatic compounds such as aromatic amines or polycyclic aromatic hydrocarbons [6]. Radiotherapy is another factor for the increased risk of developing bladder cancer in patients undergoing external-beam radiotherapy or brachytherapy [7]. Increasingly, metabolic disorders are mentioned among the important risk factors for bladder cancer development [8]. In a large prospective study, The Metabolic Syndrome and Cancer Project, Me-Can 2.0, and metabolic aberrations, especially elevated blood pressure and triglycerides, were associated with increased risks of bladder cancer among men [9]. There is increasing evidence that genetic factors and family association may influence the incidence of bladder cancer. Genome-wide association studies of bladder cancer identified several susceptibility loci [10]. However, the full pathogenesis of bladder cancer is not fully understood. Furthermore, scientific reports indicate the participation of oxidative stress in the pathogenesis of bladder cancer [11]. On the one hand, reactive oxygen species (ROS) produced as normal by-products at low to moderate levels are required for several cellular functions, but on the other hand, ROS overproduction promotes protumourigenic signaling, facilitating cancer cell proliferation, survival, and adaptation to hypoxia [12,13]. Furthermore, a high concentration of ROS causes irreversible damage to all types of cellular and extracellular macromolecules, including DNA leading to mutations [14]. In addition, ROS-sensitive signaling pathways are increased in many types of cancers, where they are involved in cellular growth, survival, proliferation, differentiation, biochemical processes, and inflammation [15,16]. Several reports describe the levels of oxidative stress markers in bladder cancer; however, there are not many reports that analyze the levels of these markers in relation to the clinical stage and the grade of the tumor.

TURBT is the standard treatment for primary bladder tumors. The purpose of TURBT is to enable histopathological diagnosis and staging, which requires the inclusion of bladder muscle in the resection specimen. According to TNM (Tumor, Node, Metastasis), classification tumors confined to the mucosa and invading the lamina propria are classified as stage Ta and T1, respectively. Flat, high-grade tumors that are confined to the mucosa are classified as carcinoma in situ (Tis). Tumors that invade muscle or perivesical tissue are classified as T2 or T3, respectively. In stage T4, the tumor invades any of the following: prostate stroma, seminal vesicles, uterus, vagina, pelvic wall, abdominal wall. Approximately 75% of patients with bladder cancer present with a disease confined to the mucosa (stage Ta, TiS) or submucosa (stage T1).

Consequently, our interest has been focused on the level of oxidative stress markers in patients with bladder cancer compared to healthy controls, as well as the association of markers with the clinical classification of cancer. Our single-center cross-sectional study aimed to determine the levels of the following markers in serum and urine in the same patients in parallel: advanced oxidation protein products (AOPP), Amadori products, total antioxidant capacity (TAC), total oxidant status (TOS), oxidative status index (OSI), and malondialdehyde (MDA). Finally, we tried to estimate the correlations between serum and urinary markers in patients with bladder cancer. To our knowledge, this is the first study to show the level of oxidative stress markers simultaneously in the serum and urine of the same patients.

## 2. Materials and Methods

### 2.1. Ethical Issues

The Bioethics Committee of the Rzeszów University approved the study protocol (2022/037 and 2022/090). All procedures performed in studies involving human participants were in accordance with the ethical standards of the institutional and/or national research committee and with the Declaration of Helsinki of 1964 and its subsequent amendments or comparable ethical standards. Written informed consent was obtained from all participants.

### 2.2. Study Group

A single-center study was carried out in a sample of 61 patients with bladder cancer and control subjects (*n* = 50). Participants were recruited from the Clinical Department of Urology and Urology Oncology of the Municipal Hospital in Rzeszow, Poland, from February to August 2022.

Inclusion criteria: the study involved newly diagnosed bladder cancer with a confirmed diagnosis based on histopathological evaluation of samples from TURBT or radical cystectomy. Histopathological evaluations of frozen sections were consistent with a urothelial bladder tumor. Patients with bladder cancer regardless of tumor grade in stage Ta-T2 were eligible for the study; patients in T3-T4 with increased uncertainty of prediction were excluded from the study. TURBTs were performed under regional anesthesia, while radical cystectomy was performed under general anesthesia using a laparoscopic transperitoneal approach. Due to the clinical stage, the participants were divided according to the TNM classification [17]. Moreover, the histological grade of the tumor was defined according to the WHO grade of 1973 [18].

**Figure 1:**
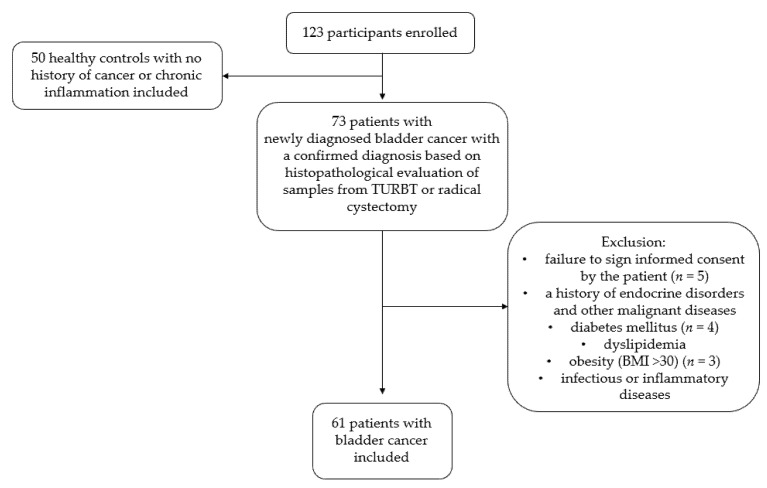
Flowchart of the study recruitment process.

Exclusion criteria included: a history of endocrine disorders and other malignant diseases, diabetes mellitus, dyslipidemia, obesity (BMI > 30), infectious or inflammatory diseases, and failure to sign informed consent by the patient. A flow chart outlining the recruitment process is shown in Figure 1.

Healthy controls were recruited among subjects who came to the local clinic to have check-ups at the same time. The control group consisted of volunteers with no history of cancer or chronic inflammation (inclusion criteria: blood counts and biochemical blood tests within the reference values; no use of any antioxidant vitamins). Control participants did not take any medication 30 days prior the study. Healthy controls had normal urinary function tests. All participants had similar socioeconomic status and similar food preferences. According to the interview conducted among the study participants, 70% of them are smokers (both in the patients and controls). Anthropometric measurements were also obtained from the participants, including height and weight. The BMI was calculated as kg/m^2^.

### 2.3. Materials

All basic reagents in analytical reagent grade were purchased from Sigma-Aldrich (Poznan, Poland). Absorptiometric measurements were made in triplicate and performed on a Tecan Infinite 200 PRO multimode reader (Tecan Group Ltd.; Männedorf, Switzerland). The results were standardized to 1 mg of total protein in the case of serum and 1 mmol of creatinine in the case of urine analysis, unless otherwise indicated.

### 2.4. Blood and Urine Collection

Blood samples were collected in the morning after fasting overnight using a Sarstedt S-Monovette closed blood collection system and placed in blood collection tubes. Next, the samples were centrifuged (1000× *g*, 10 min, 4 °C) and the serum was frozen at −80 °C until further analysis. The urine samples were collected in 50 mL sterile disposable container from the first morning portion of urine from the middle stream immediately after bedtime, centrifuged (1000× *g*, 5 min) and the supernatants were frozen at −80 °C until further analysis. The samples were stored for no longer than 120 days and thawed only once at the day of analysis.

### 2.5. Laboratory Measurements

Blood counts were analyzed using a hematology analyzer (Siemens Healthineers, Germany). Serum glucose, creatinine, and urea were analyzed using standard laboratory methods (Cobas c501, Roche Diagnostics, Mannheim, Germany). Coagulological determinations were performed on an ACL TOP 300 CTS Coagulation Analyzer (Instrumentation Laboratory, Werfen Headquarters, Barcelona, Spain). The international normalized ratio (INR) was determined using the RecombiPlasTin 2G kit from Instrumentation Laboratory and the activated partial thromboplastin time (APTT) was determined using the APTT-SP kit, also from Instrumentation Laboratory. The direct potentiometric measurement of potassium in blood serum was performed with a liquid ion-exchange electrode.

### 2.6. Biochemical Procedures

#### 2.6.1. Protein Assay

The concentration of protein in serum was determined using the standard method of Lowry et al. [19].

#### 2.6.2. Creatinine Assay

The concentration of creatinine in urine was measured using the Jaffé method [20].

#### 2.6.3. AOPP Assay

Advanced oxidation protein products (AOPP) were estimated using the method of Witko-Sarsat et al. [21]. The AOPP concentration is expressed in nanomoles of chloramine T equivalents per milligram of protein or mmol of creatinine.

#### 2.6.4. Characterization of Amadori Product by the NBT Assay

The content of the Amadori product was estimated using the method of Johnson et al. [22], with nitro blue tetrazolium. The Amadori products were estimated using an extinction coefficient of 12,640 M^−1^ cm^−1^ for monoformazan [23]. Measurements were made in duplicate.

#### 2.6.5. Total Antioxidant Capacity (TAC) Measured by Method with FRAP

FRAP was determined colorimetrically by measuring the ferric reducing capacity of the samples. Ethanol solutions with known concentrations of Trolox were used for calibration. The results were expressed in Trolox equivalents (μmol TE/L) [24].

#### 2.6.6. TAC Measured by Method with ABTS

TAC was measured by the standard method with ABTS^•^ (2,2′-azino-bis (3-ethylbenzothiazoline-6-sulphonic acid)). The results were expressed in μmol TE/L [25].

#### 2.6.7. Total Oxidant Status (TOS)

In the presence of the oxidants contained in the sample, the level of total oxidant status in the serum or urine sample was determined using the Erel method [26].

#### 2.6.8. Oxidative Stress Index (OSI)

OSI was calculated as TOS to TAC measured by method with ABTS ratio: OSI = TOS/TAC [27].

#### 2.6.9. Malondialdehyde (MDA)

The concentration of MDA in serum was determined using the standard method. The concentration of MDA in the samples was expressed as μM MDA. The results were calculated using an absorption coefficient for MDA of 1.56 × 10^5^ M^−1^ cm^−1^ [28].

### 2.7. Statistical Analysis

Data are given as mean values and standard deviations. The normality of the distribution was validated using the Shapiro-Wilk test. Statistical significance of differences was evaluated using the Kruskal-Wallis test or Mann-Whitney U test. Spearman’s rank correlation coefficient analysis was used to estimate the associations between serum and urinary markers, assuming linear dependence. Statistical analysis of the data was performed using STATISTICA software package (version 13.3, StatSoft Inc. 2017, Tulsa, OK, USA).

## 3. Results

In this study, 16 women and 45 male participants with bladder cancer were enrolled. At the same time, nine healthy women and 41 men were included into the control group. Table 1 presents the demographics and clinical laboratory results of study participants.

The bladder cancer patients were older than the controls, but the age range was similar between the two groups. BMI was similar between the study groups. The clinical laboratory results did not differ between the bladder cancer group and healthy subjects, except for a decrease in the percentage of lymphocytes and eosinophils (*p* < 0.001). The study groups had similar clotting indices. There were no differences in serum creatinine, glucose, and potassium levels, between patients and healthy controls. Bladder cancer patients had elevated serum urea levels (*p* < 0.05). More than half of the study group consisted of patients with non-invasive bladder cancer. Almost 40% of patients were diagnosed with invasive bladder cancer. Three patients were diagnosed with urothelial carcinoma in situ. The TNM staging system was used to classify the extent of cancer spread. On this basis, the patients were divided into four subgroups. Among the study participants, Ta tumor was observed in 36 people, T1 tumor in 14, T2 tumour in eight participants, whereas three cases had a TiS tumor. In addition, we used the G scale to assess cellular differentiation. A G1 tumor defined as low grade was observed in almost 40% of patients, a G2 tumor (moderate grade) in 41%, while G3 tumor (high grade) in almost 15% of the participants.

**Figure 2:**
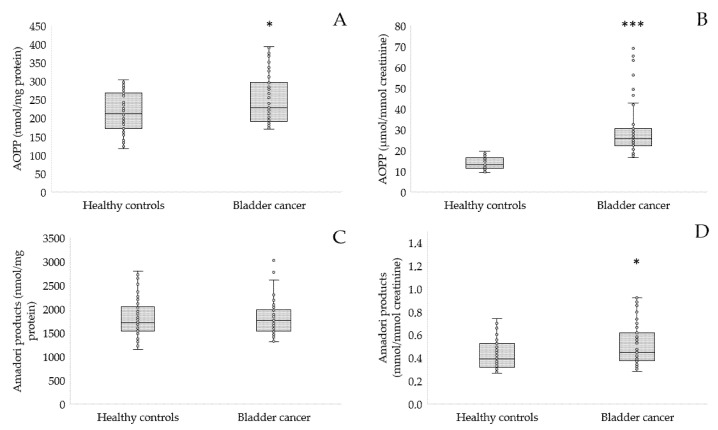
Concentration of AOPP in serum (**A**), urine (**B**) and Amadori products in serum (**C**), urine (**D**) of patients with bladder cancer in comparison to the controls; * *p* < 0.05, *** *p* < 0.001.

**Figure 3:**
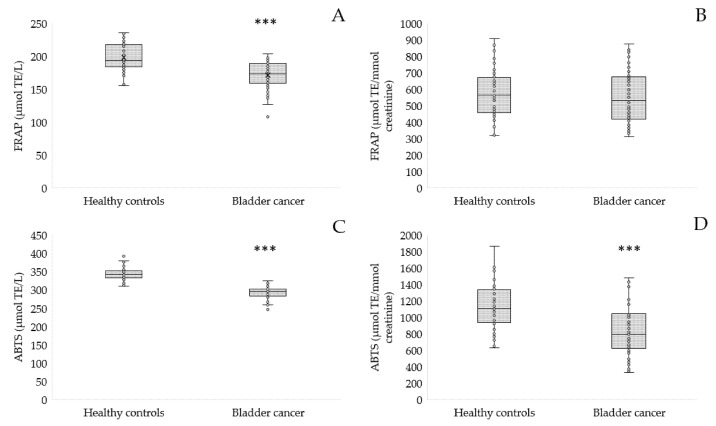
TAC measured with FRAP in serum (**A**), urine (**B**) and TAC measured with ABTS^•^ in serum (**C**), urine (**D**) of patients with bladder cancer in comparison to the controls; *** *p* < 0.001.

**Figure 4:**
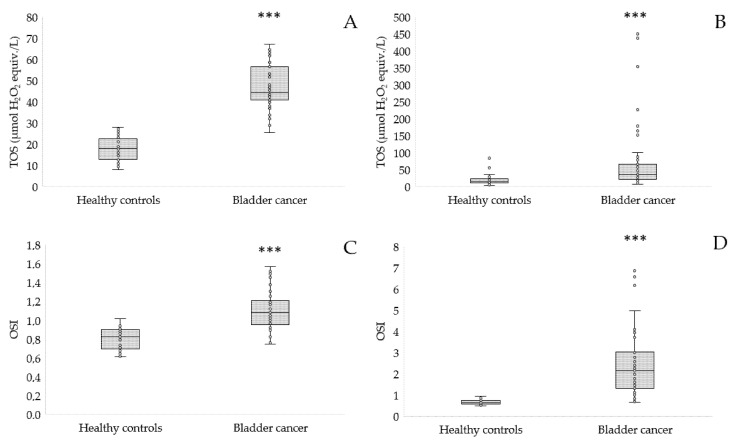
TOS in serum (**A**), urine (**B**), and OSI in serum (**C**), urine (**D**) of patients with bladder cancer in comparison to the controls; *** *p* < 0.001.

**Figure 5:**
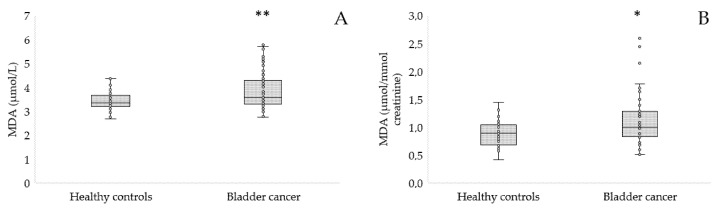
Concentration of MDA in serum (**A**) and urine (**B**) of patients with bladder cancer in comparison to the controls; * *p* < 0.05, ** *p* < 0.01.

Figure 2, Figure 3, Figure 4 and Figure 5 present levels of oxidative stress markers in patients with bladder cancer and healthy controls. In our study, we noted a statistically elevated level of AOPP, one of the most frequently estimated markers of protein oxidative modification, both in the serum (252.63 ± 67.59 vs. 215 ± 53.43 nmol/mg protein, *p* = 0.014, Figure 2A) and urine (29.47 ± 12.87 vs. 14.03 ± 2.98 μmol/mmol creatinine, *p* < 0.001, Figure 2B) of patients with bladder cancer compared to the control group. On the other hand, another protein modification, Amadori products, were at similar levels in the serum of patients and healthy participants (Figure 2C). However, the concentration of Amadori products in the urine was significantly increased in cancer patients compared to controls (*p* = 0.023, Figure 2D).

Moreover, we also assayed the TAC of serum and urine using the FRAP method and ABTS^•^ radical (Figure 3).

The TAC of the serum of the patients decreased significantly when measured using the FRAP method and ABTS^•^ (*p* < 0.001, Figure 3A,C). The TAC of the urine of the patients measured with the FRAP method was similar to healthy controls (553.61 ± 162.61 vs. 583.35 ± 147.36 μmol TE/L, Figure 3B). However, the method with ABTS^•^ revealed that the TAC of urine from bladder cancer was significantly lower than in the control group (*p* < 0.001, Figure 3D).

TOS is usually used to estimate the overall oxidation state of the body, while OSI is the ratio of TOS to TAC, which may be a more precise indicator of oxidative stress. The TOS and OSI results are shown in Figure 4.

TOS was significantly increased in both the serum and urine of patients with bladder cancer compared to healthy controls (*p* < 0.001, Figure 4A,B). Similarly, OSI was significantly higher in the bladder cancer group compared to the control group (*p* < 0.005, Figure 4C,D).

MDA belongs to the main biomarkers for assessing lipid peroxidation. The concentration of MDA in serum and urine is presented in Figure 5.

MDA was statistically elevated in both serum (3.85 ± 0.78 vs. 3.45 ± 0.42 μmol/L, *p* = 0.006, Figure 5A) and urine (1.12 ± 0.47 vs. 0.89 ± 0.23 μmol/L, *p* = 0.019, Figure 5B) of patients with bladder cancer compared to healthy controls.

Table 2 shows differences in markers of oxidative stress in bladder cancer patients, depending on the type of cancer. We found no difference in serum and urine biomarker levels between the cancer types studied.

Table 3 presents the level of biomarkers depending on the TNM classification. The TOS of the serum of patients with T2 was significantly increased compared to patients with Ta (*p* = 0.012). The level of other parameters did not differ statistically between the study groups.

Table 4 presents the level of biomarkers depending on the grading of tumor. We found that urine TAC measured by the FRAP method was statistically different between the G1 and G3 groups (*p* = 0.018). The levels of other markers did not differ statistically between the subjects divided according to the tumor classification.

Finally, we tried to assess whether serum markers were correlated with urine markers. Table 5 presents correlation coefficients and *p*-values between markers of oxidative stress in serum and urine among patients with bladder cancer.

We found a weak significant positive correlation of serum AOPP and urine AOPP. Furthermore, the TAC measured by FRAP in serum was correlated with the TAC measured by FRAP in urine. Additionally, serum TOS was inversely correlated with urine TAC measured by FRAP and ABTS^•^ and positively correlated with urine TOS, OSI, and MDA. Similarly, serum OSI was inversely correlated with urine TAC measured by FRAP and ABTS^•^ and positively correlated with urine TOS and OSI. Interestingly, there was no correlation between the urine markers of oxidative stress and serum Amadori products, TAC measured by ABTS^•^, and MDA.

## 4. Discussion

The influence of oxidative stress in the complex pathophysiology of urogenital cancers is accepted [29,30,31], where the inflammatory and oxidative pathways played an important role. Moreover, the metabolism of many compounds, such as tobacco smoke or other environmental chemicals, leads under certain conditions to ROS formation [32]. If the body is exposed to large amounts or the prolonged action of exogenous ROS-generating substances, cellular structures and processes can be damaged, causing serious disorders that could later initiate an oncogenesis [33,34]. ROS experimentally induce a long interspersed expression of nuclear element-1 encoded protein expression and promote migration in bladder cancer cells [35]. The use of oxidative stress biomarkers and the oxidative stress-related gene signature to guide treatment decisions along with the development of immunotherapy may benefit the patient [36]. Furthermore, the increase in ROS may be associated with impairment in the microenvironment of cancer cells [37].

The main aim of the study was to investigate differences in the level of serum and urine oxidative stress markers in bladder cancer in a different tumor types, stages, and grade.

The main findings of our study are elevated levels of oxidative stress markers and decreased antioxidant capacity in the serum and urine of bladder cancer compared to controls. Furthermore, we did not observe associations between the level of markers of oxidative stress and the type of tumor, clinical stage, and grade.

In our study, AOPP, which belongs to markers of protein oxidation, was significantly elevated in serum and urine from bladder cancer. Significantly higher values for AOPP in the studied group with bladder cancer than in the control (279.96 ± 246.86 vs. 119.26 ± 53.24 μmol/L, *p* < 0.01) were seen in the study by Sawicka et al. [38]. Likewise, other markers of protein damage—protein carbonyl groups were higher, while thiol groups were significantly lower in bladder cancer patients than in healthy controls [39,40,41]. To our knowledge, our study of Amadori product is the first to report the levels in the serum and urine of bladder cancer. Amadori products, which are formed in the glycation process, generate ROS such as superoxide anions [42]. In the current study, the levels of Amadori products were elevated in the serum and urine of patients with bladder cancer patients.

In our study, the TAC as expressed by the ability to neutralize radicals was decreased in both serum and urine in patients with bladder cancer compared to controls. Similar results of reduced antioxidant capacity of the plasma of bladder cancer patients as determined by the radical DPPH^•^ and ABTS^•^ have been described previously [38,40,43].

Tests for serum and urine oxidative stress parameters showed that the bladder cancer patient group had higher TOS and OSI values; similar results were previously observed in ref. [44]. The increased level of TOS may be associated with a decrease in antioxidant enzyme activity and an increase in the xanthine oxidase activity, which is involved in the generation of ROS in bladder cancer [41,45]. Moreover, the glutathione peroxidase, which belongs to antioxidant enzymes, can significantly increase the risk of bladder cancer and it may further affect the disease status of bladder cancer [46].

MDA is the major aldehyde end product of the lipid peroxidation of membrane fatty acids by free radicals [47]. We found an elevated level from MDA in the serum and urine of patients with bladder cancer. Similarly, MDA was even three times higher in plasma samples from cancer patients, compared to recent control group in the studies published [38,40,43]. Urinary MDA was also elevated in patients with bladder cancer compared to controls (9.54 ± 8.65 vs. 6.76 ± 5.83 μmol/g creatinine, *p* < 0.05) [48]. In addition, other markers of lipid peroxidation have been studied and found to increase in bladder cancer tissue, such as 4-hydroxynonenal, suggesting that lipid, including membrane phospholipids, alongside proteins, are the main target of ROS in bladder epithelium cells [35]. 

There were three types of cancer among the participants in our study. We did not observe any statistical difference in the level of biomarkers of oxidative stress between the study groups. Therefore, it seems that the severity of oxidative stress is not related to the type of cancer.

The next step of our study was to determine the markers of oxidative stress according to the clinical stage of the cancer. Apart from TOS, we did not observe any statistical difference in the markers level of the tested depending on the clinical stage of the cancer. However, the AOPP concentration was decreased in the Ta stage compared with T1, T2, and TiS stages, but the differences were not statistically significant. Contrary to our results, the concentration of AOPP was reduced at T2 stage when compared to the Ta and T1 stages (151.9 vs. 252 and 258.1 μmol/L) [38].

The serum concentrations of MDA were significantly increased for patients in all stages in comparison to the control in the study by Sawicka et al. [38]. However, similarly to our study, no differences were observed in the median MDA level between Ta, T1, and T2 stages. On the other hand, the MDA level in the T1 and T2-T4 group was significantly elevated than the MDA level in the Ta group. However, no significant differences were observed in the MDA level between the T1 and T2-T4 groups [49]. In contrast to our results, the more invasive stages of bladder cancer with greater progression potential (T2-T4) were related to increased enzyme antioxidant activity and decreased non-enzymatic antioxidant capacity [50]. In some cases, especially in the early stages, cancer cells could adapt to intrinsic oxidative stress or drug-induced oxidative stress by increasing their antioxidant system [51]. 

Regarding the distribution of study participants according to tumor grade, we did not see any differences other than urine TAC, which was reduced in the G3 group compared to the G1 group. We did not observe an increasing concentration of oxidative stress markers with increasing clinical tumor grade. Interestingly, a decrease in AOPP concentration together with an increase in cancer grade in recent study [38].

We did not find a statistical difference in the level of MDA between the study groups divided by the clinical grade of cancer. However, MDA was elevated in the G2 and G3 groups compared to G1 [38]. However, in contrast to our results, a recent study showed significant differences between the TaG1 grade, and the T1G2 grade, which demonstrated a positive correlation between the degree of development of bladder cancer and the level of oxidative stress [40]. The lack of significant differences in marker level between the groups in our study may be due to the small number of participants.

Finally, we tested correlations between the serum and urinary markers of oxidative stress. We found several correlations, especially between serum TOS, and OSI and urine TAC, TOS, and OSI. The urine biomarkers level represents the intergraded indicators of redox balance over a longer period of time compared to serum levels [52].

The regulation of oxidative stress is an important mechanism participated in tumor development, but also in the effectiveness of anticancer treatment. ROS may induce several different cellular or molecular targets, such as growth inhibition, cell division, and death, in a concentration-dependent manner, suggesting new therapeutic approaches for the treatment of bladder cancer [53].

Molecular biomarkers, such as those adopted in the current study, are an emerging and promising tool. However, prospective validation studies are needed to adopt reliable prognostic markers in daily clinical practice for patients with bladder cancer [54]. Furthermore, several studies suggest that hypercoagulability, inflammation, and malnutrition promote the occurrence, development, recurrence, and metastasis of different tumors and are associated with a poorer postoperative course and worse oncological outcomes [55,56,57,58]. Recent findings suggest that the control of the nutritional status score and the preoperative albumin to fibrinogen ratio could be prognostic values in patients with bladder cancer treated with radical cystectomy [55,57].

Nevertheless, this study has some limitations that should be mentioned. First, the study is a single-center study and includes a limited number of participants, especially in the group of patients with urothelial carcinoma in situ. The control group was slightly younger. In addition, we did not measure serum and urinary antioxidant levels. Our study also has strengths—A well-characterized group of patients in clinical terms, in whom markers of oxidative stress in serum and urine were simultaneously evaluated. Moreover, so far there are not many reports on the determination of oxidative stress markers in the urine of patients with bladder cancer.

## 5. Conclusions

In conclusion, our study shows a significant increase in the levels of oxidative stress markers with a decrease in serum and urine in patients with bladder cancer compared to healthy people. However, we observed virtually no difference in the level of markers of oxidative stress depending on the type of cancer, its clinical stage and grade. Furthermore, the biomarkers, especially TOS and OSI, evaluated in serum and urine, correlated with each other. Taken together, our results indicate the participation of oxidative stress in the development of bladder cancer. However, the contribution of oxidative stress to tumor progression should be investigated in a larger number of participants before biochemical markers of oxidative stress find clinical application in the assessment of bladder cancer progression. From a clinical perspective, it seems that further studies should also consider antioxidant therapy in patients with bladder cancer.

## Figures and Tables

**Table 1:** Participant demographics at enrolment *.

—		Healthy Control	Bladder Cancer	*p*
*n*		50	61	
F/M		wrz.41	16/45	
Age (years)	mean ± SD	63.28 ± 11.74	71.75 ± 9.2	<0.001
	range	44–90	40–91	
BMI (kg/m^2^)	mean ± SD	26.39 ± 3	26.22 ± 3.06	0.904
	range	19.05–29.66	19.72–29.91	
WBC (10^3^/µL)	mean ± SD	7.05 ± 1.5	7.21 ± 2.48	0.518
	range	4.2–10	2.8–17.5	
LYM (%)	mean ± SD	29.58 ± 4.36	24.79 ± 8.32	<0.001
	range	25.1–38.9	5.7–42.2	
MONO (%)	mean ± SD	7.49 ± 1.78	8.56 ± 3.58	0.561
	range	3.3–10	2.3–24.7	
NEU (%)	mean ± SD	63.12 ± 6.44	64.24 ± 9.79	0.895
	range	50.3–70	42.8–86.8	
EOS (%)	mean ± SD	1.98 ± 1.25	1.47 ± 1.4	<0.001
	range	1–4.9	0–7.8	
BASO (%)	mean ± SD	0.57 ± 0.31	0.5 ± 0.29	0.091
	range	0.2–1.7	0.1–1.6	
Prothrombin time (s)	mean ± SD	11.75 ± 0.53	12.15 ± 1	0.694
	range	10.4–12.5	10.4–14.9	
Prothrombin time (%)	mean ± SD	94.52 ± 10.58	93.86 ± 11.37	0.777
	range	80–119	68–119	
INR	mean ± SD	1.05 ± 0.08	1.05 ± 0.08	0.435
	range	0.9–1.2	0.9–1.3	
APTT (s)	mean ± SD	29.6 ± 3.47	30.84 ± 6.71	0.784
	range	25.4–36.8	22.7–57.8	
Creatinine (mg/dL)	mean ± SD	0.88 ± 0.17	1.04 ± 0.5	0.938
	range	0.6–1.17	0.52–3.39	
Glucose (mg/dL)	mean ± SD	96.81 ± 6.58	100.07 ± 5.79	0.077
	range	85–106	84–109	
Urea (mg/dL)	mean ± SD	33.09 ± 8.85	41.07 ± 16.98	0.045
	range	16–49	16–114	
K^+^ (mmol/L)	mean ± SD	4.33 ± 0.28	4.37 ± 0.44	0.747
	range	3.7–5	3.4–5.8	
Types of cancer				
non-invasive urothelial carcinoma	*n* (%)	-	36 (59)	-
invasive urothelial carcinoma	*n* (%)	-	22 (36)	-
urothelial carcinoma *in situ*	*n* (%)	-	3 (5)	-
Clinical stage (TNM)				
Ta	*n* (%)	-	36 (59)	-
T1	*n* (%)	-	14 (23)	-
T2	*n* (%)	-	8 (13)	-
TiS	*n* (%)	-	3 (5)	-
Clinical grade				
G1	*n* (%)	-	24 (39.3)	-
G2	*n* (%)	-	25 (41)	-
G3	*n* (%)	-	9 (14.7)	-

* BMI—body mass index, WBC—white blood cells, LYM—lymphocytes, MONO—monocytes, NEU—neutrophils, EOS—eosinophils, BASO—basophils, INR—international normalized ratio, APTT—activated partial thromboplastin time, TNM—classification of malignant tumors (Tumor, Node, Metastasis): Ta—non-invasive papillary tumor; T1—tumors invasive subepithelial connective tissue; T2—tumors invading the muscle of bladder wall; TiS—urothelial carcinoma in situ; G1—low grade, G2—moderately grade, G3—high grade.

**Table 2:** Markers of oxidative stress in participants with bladder cancer depending on type of cancer *.

		Non-Invasive Urothelial Carcinoma	Invasive Urothelial Carcinoma	Urothelial Carcinoma In Situ	*p*
Serum	AOPP (nmol/mg protein)	232.89 ± 71.53	246.35 ± 69.22	259.21 ± 46.01	0.787
	Amadori products (nmol/mg protein)	1683.4 ± 191.07	1833.98 ± 374.58	1826.09 ± 342.45	0.736
	FRAP (μmol TE/L)	175.61 ± 8.26	176.39 ± 18.85	167.34 ± 23.44	0.718
	ABTS (μmol TE/L)	291.96 ± 15.27	293.99 ± 18.49	296.71 ± 11.27	0.354
	TOS (μmol H_2_O_2_ equiv./L)	37.12 ± 6.1	44.19 ± 10.39	50.99 ± 19.78	0.171
	OSI	1.06 ± 0.15	1.07 ± 0.16	1.16 ± 0.03	0.337
	MDA (μmol/L)	3.2 ± 0.32	3.77 ± 0.74	3.97 ± 0.82	0.165
Urine	AOPP (μmol/mmol creatinine)	28.51 ± 12.41	29.23 ± 12.71	35.19 ± 18.72	0.643
	Amadori products (mmol/mmol creatinine)	0.53 ± 0.17	0.49 ± 0.21	0.53 ± 0.08	0.169
	FRAP (μmol TE/mmol creatinine)	573.1 ± 169.08	492.59 ± 136.78	666.24 ± 173.53	0.253
	ABTS (μmol TE/mmol creatinine)	830.71 ± 281.73	788.73 ± 294.05	883.72 ± 185.12	0.806
	TOS (μmol H_2_O_2_ equiv./L)	67.19 ± 93.11	72.86 ± 95.04	26.16 ± 7.13	0.285
	OSI	2.06 ± 1.66	2.16 ± 1.12	2.65 ± 1.65	0.388
	MDA (μmol/mmol creatinine)	0.89 ± 0.31	1.08 ± 0.44	1.17 ± 0.5	0.495

* AOPP—advanced oxidation protein products, FRAP—total antioxidant capacity measured by FRAP, ABTS—total antioxidant capacity measured by ABTS^•^, TOS—total oxidant status, OSI—oxidative status index, MDA—malondialdehyde.

**Table 3:** Markers of oxidative stress in participants with bladder cancer depending on the TNM classification *.

		Ta	T1	T2	TiS	*p*
Serum	AOPP (nmol/mg protein)	232.89 ± 71.53	246.85 ± 67.02	248.77 ± 67.76	259.21 ± 46.01	0.752
	Amadori products (nmol/mg protein)	1683.4 ± 191.07	1717.01 ± 189.5	1817.82 ± 308.1	1826.09 ± 342.45	0.69
	FRAP (μmol TE/L)	175.61 ± 8.26	177.6 ± 20.65	173.46 ± 18.11	167.34 ± 23.44	0.857
	ABTS (μmol TE/L)	291.96 ± 15.27	296.09 ± 14.79	286.65 ± 24.75	296.71 ± 11.27	0.734
	TOS (μmol H_2_O_2_ equiv./L)	37.12 ± 6.1	48.52 ± 11.85	48.11 ± 9.1	50.99 ± 19.78	0.017
	OSI	1.06 ± 0.15	1.11 ± 0.18	1.09 ± 0.2	1.16 ± 0.03	0.504
	MDA (μmol/L)	3.2 ± 0.32	4.18 ± 0.91	3.63 ± 0.41	3.97 ± 0.82	0.22
Urine	AOPP (μmol/mmol creatinine)	28.51 ± 12.41	31.08 ± 13.7	30.61 ± 15.58	35.19 ± 18.72	0.728
	Amadori products (mmol/mmol creatinine)	0.53 ± 0.17	0.61 ± 0.25	0.4 ± 0.05	0.53 ± 0.08	0.381
	FRAP (μmol TE/mmol creatinine)	573.1 ± 169.08	585.23 ± 176.33	430.01 ± 31.11	666.24 ± 173.53	0.127
	ABTS (μmol TE/mmol creatinine)	830.71 ± 281.73	921.35 ± 385.57	751.94 ± 225.08	883.72 ± 185.12	0.722
	TOS (μmol H_2_O_2_ equiv./L)	67.19 ± 93.11	38.97 ± 23.19	119.81 ± 148.98	26.16 ± 7.13	0.345
	OSI	2.06 ± 1.66	2.1 ± 1.35	2.49 ± 1.7	2.65 ± 1.65	0.52
	MDA (μmol/mmol creatinine)	0.89 ± 0.31	1.12 ± 0.43	1.1 ± 0.26	1.17 ± 0.5	0.703

* AOPP—advanced oxidation protein products, FRAP—total antioxidant capacity measured by FRAP, ABTS—total antioxidant capacity measured by ABTS^•^, TOS—total oxidant status, OSI—oxidative status index, MDA—malondialdehyde; Ta—non-invasive papillary tumor; T1 = tumors invasive subepithelial connective tissue; T2—tumors invading the muscle of bladder wall; TiS—urothelial carcinoma in situ.

**Table 4:** Markers of oxidative stress in participants with bladder cancer depending on the cancer grading *.

		G1	G2	G3	*p*
Serum	AOPP (nmol/mg protein)	247.21 ± 66.23	262.72 ± 74.84	244.14 ± 67.17	0.974
	Amadori products (nmol/mg protein)	1788.16 ± 221.48	1828.79 ± 411.54	1788.16 ± 221.48	0.381
	FRAP (μmol TE/L)	181.1 ± 16.62	169.74 ± 23.17	166.51 ± 21.96	0.177
	ABTS (μmol TE/L)	291.84 ± 14.89	290.05 ± 17.98	293.27 ± 15.61	0.707
	TOS (μmol H_2_O_2_ equiv./L)	42 ± 14.2	47.27 ± 8.14	47.93 ± 8.87	0.518
	OSI	1.09 ± 0.16	1.1 ± 0.2	1.18 ± 0.18	0.745
	MDA (μmol/L)	3.76 ± 0.68	3.93 ± 0.82	3.93 ± 0.86	0.883
Urine	AOPP (μmol/mmol creatinine)	30.26 ± 13.87	27.04 ± 7.58	33.73 ± 20.41	0.893
	Amadori products (mmol/mmol creatinine)	0.47 ± 0.16	0.56 ± 0.19	0.52 ± 0.25	0.235
	FRAP (μmol TE/mmol creatinine)	619.49 ± 163.97	525.79 ± 135.3	479.77 ± 151.18	0.022
	ABTS (μmol TE/mmol creatinine)	856.56 ± 288.46	802.9 ± 296.94	774.74 ± 320.23	0.583
	TOS (μmol H_2_O_2_ equiv./L)	66.91 ± 76.25	68.28 ± 96.73	97.7 ± 144.72	0.865
	OSI	1.8 ± 1.48	2.1 ± 1.41	3.03 ± 1.5	0.075
	MDA (μmol/mmol creatinine)	1.15 ± 0.4	1.15 ± 0.5	1.16 ± 0.61	0.754

* AOPP—advanced oxidation protein products, FRAP—total antioxidant capacity measured by FRAP, ABTS—total antioxidant capacity measured by ABTS•, TOS—total oxidant status, OSI—oxidative status index, MDA—malondialdehyde; G1—low grade, G2—moderately grade, G3—high grade.

**Table 5:** Spearman’s rank correlation coefficients and *p* values between oxidative stress parameters in serum and urine *.

		Serum													
		AOPP		Amadori Products		FRAP		ABTS		TOS		OSI		MDA	
		R	*p*	R	*p*	R	*p*	R	*p*	R	*p*	R	*p*	R	*p*
Urine	AOPP	0.272	0.038	0.231	0.081	−0.032	0.811	−0.078	0.556	0.146	0.274	−0.032	0.0492	0.072	0.591
	Amadori products	−0.03	0.827	0.152	0.262	0.094	0.49	0.068	0.617	0.258	0.054	0.091	0.507	−0.001	0.992
	FRAP	0.088	0.52	0.26	0.052	0.286	0.036	0.173	0.201	−0.294	0.027	−0.365	0.006	0.115	0.398
	ABTS	−0.107	0.431	0.046	0.737	−0.118	0.386	0.231	0.067	−0.0383	0.003	−0.285	0.036	0.053	0.697
	TOS	−0.001	0.994	−0.086	0.515	0.156	0.231	−0.073	0.583	0.418	0.001	0.45	0.001	0.045	0.748
	OSI	−0.065	0.637	−0.246	0.073	0.023	0.866	−0.208	0.13	0.291	0.032	0.339	0.012	0.042	0.754
	MDA	−0.085	0.532	−0.084	0.539	0.001	0.997	0.072	0.597	0.271	0.048	0.182	0.179	0.165	0.223

* AOPP—advanced oxidation protein products, FRAP—total antioxidant capacity measured by FRAP, ABTS—total antioxidant capacity measured by ABTS^•^, TOS—total oxidant status, OSI—oxidative status index, MDA—malondialdehyde.

## Data Availability

The datasets generated during and/or analyzed during the current study are available from the corresponding author on reasonable request.
